# Women Who Emerge as Leaders in Temporarily Assigned Work Groups: Attractive and Socially Competent but Not Babyfaced or Naïve?

**DOI:** 10.3389/fpsyg.2018.02553

**Published:** 2018-12-19

**Authors:** Freya M. Gruber, Carina Veidt, Tuulia M. Ortner

**Affiliations:** Department of Psychology, University of Salzburg, Salzburg, Austria

**Keywords:** leadership emergence, babyfacedness, attractiveness, social competence, naïveté

## Abstract

The underrepresentation of women in top positions has been in the spotlight of research for decades. Prejudice toward female leaders, which decreases women’s chances of emerging as leaders, has been discussed as a potential reason. Aiming to investigate the underlying mechanisms of this prejudice, we focused on the question of how facial characteristics might influence women’s leadership emergence. Because other research has related ascribed social competence and ascribed naïveté to attractiveness and babyfacedness, respectively, we hypothesized that ascribed social competence would mediate the impact of ascribed attractiveness on leadership emergence and that ascribed naïveté would mediate the impact of ascribed babyfacedness on leadership emergence. In a pilot study, we analyzed data from 101 participants of a women’s leadership contest held in 2015 in Germany. We then confirmed these results in a methodologically improved main study on other women who participated in the contest in one of two other years: 2016 and 2017 (*N* = 195). Women applied to participate in the contest by recording their answers to several questions in a video interview. In the contest, they were assigned to teams of about ten women each and worked on several assessment-center-like tasks. After each task, each member of each team nominated the three women they believed showed the best leadership potential in their group. We operationalized women’s leadership emergence as the number of nominations received. We measured participants’ facial attractiveness, babyfacedness, social competence, and naïveté by having raters follow a specifically developed rating manual to rate the answers the women gave in the video interviews. In both studies, the results indicated that women with higher ascribed facial attractiveness had higher ascribed social competence, which significantly predicted leadership emergence in the contest. Likewise, women with higher ascribed babyfacedness had higher ascribed naïveté, which significantly, albeit only slightly, negatively predicted leadership emergence. We discuss the implications of the results for personnel selection.

## Introduction

In modern Western societies, sex-related barriers to occupational success still exist and are reflected in economic data. Women hold only 28% of the CEO positions in the United States (U.S. Bureau of Labor Statistics, 2017) and 16% of the 1,500 board seats of the S&P (Ernst and Young LLP, 2015). Women occupy fewer top executive positions than men in the European Union (Bourgeais, 2017) and fewer full professorships worldwide (Johnson, 2017). Because a lack of qualified women can no longer explain these circumstances (European Commission, 2012; Johnson, 2017), it is important to ask why women rarely hold these top leadership positions. One possible explanation for this inequity can be found in differences in leadership emergence. Taggar et al. (1999) defined leadership emergence as the process by which one team member becomes the leader of an initially leaderless group without holding the position by title or by being previously assigned to it. According to Paunova (2015), one or more leaders may emerge within a group when they are perceived and acknowledged as a leader, for instance, due to the positive effect of their leader behavior on the group during a task (Bass and Bass, 2008). Systematic gender differences in leadership emergence may create obstacles for women because being perceived as not able to emerge as a leader may prevent them from being hired or promoted (e.g., Eagly and Karau, 1991, 2002; Finkelstein et al., 2018). Therefore, it is important to further investigate and understand the underlying mechanisms that can help women emerge as leaders or prevent them from doing so.

Scientists have formulated several theories to explain differences in perceptions of the leadership potential of women and men and differences in their leadership emergence: As a basic principle, social role theory (Eagly, 1987) suggests that differences in the behavior of women and men and also differences in how women and men are perceived in a society stem primarily from the different distributions of men’s and women’s social roles. In this theory, different roles occupied by men and women have an impact on sex-related stereotypes and ascribed aspects of personality because people tend to make inferences from these roles to associated characteristics. In fact, various studies of different fields have demonstrated relations between social roles and the ascribed characteristics of people (e.g., Diekman and Schneider, 2010; Ortner et al., 2011; Koenig and Eagly, 2014). Following this reasoning, Eagly and Karau (2002) formulated role congruity theory to further explain women’s greater difficulties in reaching leadership positions. According to this theory, individuals receive positive evaluations by others when their characteristics are estimated as confirming the group’s typical social roles (Eagly and Diekman, 2005). In fact, “many people perceive [incongruity] between the characteristics of women and the requirements of leader roles” (Eagly and Karau, 2002, p. 574). This perceived incompatibility between stereotypes of a social group and the requirements for fulfilling a specific role is supposed to result in prejudice, backlash effects (Rudman, 1998), and low evaluations of women as actual or potential leaders (see Eagly and Karau, 2002).

Another related, albeit more general theory that has contributed to explaining inequities in society on the basis of social group processes is the expectation states theory (Wagner and Berger, 1997; Weyer, 2007). This theory addresses small, task-oriented work groups and highlights the relevance of status characteristics (e.g., gender, age, ethnicity) but also physical attributes for the ascription of task-relevant characteristics. Carli and Eagly (2001) employed this approach to explain differences in the leadership emergence of men and women, proposing that there are biases in evaluations of female leaders. Ridgeway (2001) described this approach as applying expectation states theory in a new context. Accordingly, gender roles are enrooted in the social hierarchy and in leadership because the core of gender stereotypes are cross-cultural schemas about women’s status positions, also called status beliefs. These status beliefs have been proposed to be a basic reason for lower leadership emergence in women than in men because men, rather than women, are associated with general competence and status worthiness. However, a recent analysis integrating 16 different opinion polls revealed an increase for women’s ascribed competence over the past years and concluded gender equality in ascribed competence for women and men in the United States (see Eagly et al., 2018).

Nevertheless, several studies have provided empirical support for the validity of these theoretical frameworks in explaining differences in the performances of women and men with reference to leadership: For example, Garcia-Retamero and López-Zafra (2006) conducted an experiment to test how prejudice against female leaders in different work environments stems from people’s expectations. To do so, they asked non-leader participants to rate vignettes of hypothetical candidates for a leadership position. The data indicated prejudice against women except when the woman worked “in an industry congruent with her gender role” (p. 58). Further results indicated effects of social attribution by illustrating that incongruence between masculine task demands and the female gender role reduced leadership emergence even in dominant women (see Ritter and Yoder, 2004).

A more complex and new model combining aspects of personality traits with behavioral mechanisms was recently proposed along with a meta-analysis on leadership emergence: Badura et al. (2018) addressed the question of why men more frequently emerge as leaders than women. For this purpose, the authors proposed gender, individual characteristics, behavioral aspects, as well as situational factors as determinants of the relation between gender and leadership emergence. In order to test their gender-agency/communion-participation (GAP) model, they coded 1,632 effect sizes. Results indicated a beneficial effect of people’s gender on leadership emergence through agency (e.g., assertiveness and dominance), but a detrimental effect through communion (e.g., kindness and nurturance). Furthermore, the relations between these traits and leadership emergence were mediated through participative behavior in group discussions. Moreover, they hypothesized that gender differences in leadership emergence depended on situational factors such as the study’s setting (business settings, lab settings, classroom settings), gender egalitarianism in the nation where the data were collected, the length of interaction, and the degree of social complexity of the task. Their results indicated that the gender gap in leadership emergence was significantly lower in business settings compared with lab settings and significantly larger when the interaction time in the task was shorter.

Besides the influence of agency, communion, and behavioral aspects, physical appearance may also contribute to leadership emergence. Much research has revealed that a person’s physical appearance is a relevant characteristic in various areas of life, for example, partner selection (Langlois et al., 2000; for a review, see Rhodes, 2006), political elections (Olivola and Todorov, 2010), judicial decisions (Efran, 1974; Zebrowitz and McDonald, 1991), decisions regarding personnel selection (Zebrowitz et al., 1991; Shannon and Stark, 2003), and also ascribed leadership competences (Sczesny and Kühnen, 2004) or leadership selection processes (Stoker et al., 2016). Attractiveness represents one of the most salient aspects of physical appearance and, therefore, affects the ways in which individuals perceive and evaluate others (Little et al., 2011). Several theoretical explanations have also been proposed to account for the relevance of physical attractiveness in the field of leadership: Implicit personality theory by Bruner and Tagiuri (1954) postulated that people unconsciously build hypothetical constructs of trait elements and inferential relations between these attributes when assessing other people (see Schneider, 1973). On the basis of their meta-analysis of 76 studies, Eagly et al. (1991) identified the association between “beauty” and “goodness” as a physical attractiveness stereotype. They reported a significantly higher attribution of favorable characteristics to attractive individuals in comparison with unattractive individuals. Highly relevant for the domain of leadership emergence, individuals rated as attractive received higher evaluations with reference to social competence, adjustment, potency, and intellectual competence. This finding is in line with a study that reported findings that individuals perceived targets whose faces they had rated as attractive with respect to facial symmetry and proportions as more successful, satisfied, and likable than people whose faces they had rated as unattractive (Braun et al., 2001). In fact, studies have revealed positive effects of ascribed attractiveness on life and business outcomes such as career success and management level (Dietl, 2013), income (Judge et al., 2009), and also ascribed leadership status (Cherulnik et al., 1990). According to Cherulnik (1995), the positive impact of facial attractiveness on leader emergence even accumulates over a lifetime because others’ attributions create a favorable distinctive social environment that provides special experiences and opportunities. Individuals judged as attractive were also found to be more confident in their social skills than individuals judged as unattractive (Cherulnik, 1995). Moreover, Sobieraj and Krämer (2014) demonstrated a strong relation between ascribed social competence and ascribed physical attractiveness in a study with experimentally varied virtual avatars. On the basis of these findings, we hypothesized that *ascribed facial attractiveness would positively predict leadership emergence (Hypothesis 1)*. Moreover, we hypothesized that *social competence would positively mediate the impact of facial attractiveness on leadership emergence (Hypothesis 2)* because social competence represents a determinant of leadership skills (Riggio et al., 2003; Groves, 2005; Riggio and Reichard, 2008).

However, not only facial attractiveness affects the ways in which individuals perceive and describe others. The anatomy literature indicates sex differences in facial features (e.g., Enlow, 1982; Gray and Standring, 2008) with female faces having more in common with infantile, babyface-like facets compared with male faces (Guthrie, 1976). Lorenz (1943) defined this set of immature facial characteristics (e.g., big round eyes, a big head, a round face, or a small nose) as the so-called baby schema (German: “Kindchenschema”) or babyfacedness. In an evolutionary approach, Perrett et al. (1998) attributed the preference of humans for feminine facial features to the correlations of estrogen-dependent traits with aspects of health and reproductive fitness. Nevertheless, the costs of this form of attractiveness were addressed by investigating whether the more babyish faces of women compared with men further contribute to the attribution of sex-related characteristics and stereotypes. Presenting equally mature-faced male and female faces weakened stereotyped ascriptions and led to the conclusion that women’s average facial features promote ascriptions of stereotyped characteristics (Friedman and Zebrowitz, 1992).

In line with these findings, Eagly and Karau’s (2002) role congruity theory provides a framework that can be applied to formulate hypotheses on the effects of babyfacedness on leadership emergence: Whereas the leader role is predominantly characterized by agentic traits, such as being competitive, self-confident, aggressive, objective, and ambitious (see Koenig et al., 2011), people possessing a babyfaced physiognomy were described as less physically strong, less socially dominant, less astute (Zebrowitz McArthur and Apatow, 1983/1984), more naïve, and weaker compared with people possessing more mature faces (Keating et al., 2003; for a review, see Montepare and Zebrowitz, 1998). According to these findings, possessing a babyface is inconsistent with the characteristics required by a leadership role. A growing number of studies focusing on babyfacedness have supported this assumption: For instance, studies have indicated a negative relation between babyfacedness and inferred competence among politicians (Poutvaara et al., 2009), and a negative relation between babyfacedness and leadership status in a high school senior class (Cherulnik et al., 1990). Furthermore, Cherulnik (1995) found in an experiment that facial maturity was a significant predictor of leadership emergence. Investigations of hiring preferences have revealed similar findings (Zebrowitz et al., 1991): Undergraduate participants preferred male and mature-faced applicant targets for leadership positions over female and babyfaced applicants, especially for higher-status positions.

It seems plausible that a person with a high level of ascribed naïveté (defined as a collective term that summarizes how naïve, inexperienced, credulous, and generally intelligent an individual is perceived to be) due to his or her facial appearance would find it difficult to meet leader role expectations. Women may be particularly inclined to suffer from this misperception because they are more likely to encounter prejudice due to role incongruence in the first place (Eagly and Karau, 2002). In addition, women are more likely to be judged as babyfaced (Friedman and Zebrowitz, 1992; Chiao et al., 2008; Olivola and Todorov, 2010). In the second part of this study, we therefore hypothesized that *ascribed babyfacedness would negatively predict leadership emergence (Hypothesis 3).* Moreover and analogous to Hypothesis 2, we investigated whether these babyface-specific attributions could explain this negative association: Therefore, we hypothesized that *ascribed naïveté would negatively mediate the impact of ascribed babyfacedness on leadership emergence (Hypothesis 4)*.

To summarize the aims of the present study, we intended to investigate the impact of facial characteristics on women’s chances of emerging as a leader, and further, whether the ascription of other attributes that are based on facial characteristics would mediate this effect in the setting of a women’s leadership contest. On the one hand, we hypothesized that ascribed attractiveness would be a positive predictor of leadership emergence and that ascribed social competence would mediate this relation. On the other hand, we hypothesized that ascribed babyfacedness would be a negative predictor of leadership emergence and that the ascription of naïveté would mediate this relation. Data collected during contests that were held exclusively for women in three consecutive years provided a basis of the analyses: 2015–2017. The predictor variables (ascribed attractiveness and ascribed babyfacedness) and the mediator variables (ascribed social competence and ascribed naïveté) comprised raters’ evaluations of women’s appearance in video interviews from self-recorded videos from the contest application phase. Peer nominations provided by group members after participating in assessment-center-like tasks at the contests served as the dependent variable leadership emergence. Therefore, we present a pilot study, which uses data from the 2015 contest, and then the main study containing a larger sample (data from the 2016 and 2017 contests) and a revised method.

## General Methods

The analyses based on data collected across three consecutive years of a women’s leadership contest conducted in Germany.^1^ Data collection took place in two phases: In the a*pplication phase*, women applied to participate in the contests by submitting their curriculum vitae and letters of recommendation as well as by using a professional software platform to record a video interview consisting of their answers to five or six questions. For each question, applicants could prepare their answer within 2 min. Moreover, participants were instructed to record each of their responses in a maximal duration of 3 min. The women who passed this application phase on the basis of the quality and persuasive power of all of their application documents were invited to participate in the actual leadership contest. At *the leadership contest* – between attendance at keynote presentations, workshops, and networking slots – contest participants took part in 2 to 3 assessment-center-like tasks in which they engaged in group tasks or group discussions or prepared speeches in groups of 7 to 12 women each. After each task, the women nominated the three group members who had been most convincing as leaders. Contest participants who received the most nominations by their peers were rewarded with vouchers, for example, for travel tickets or career coaching. Participants’ data were included in our study only when they agreed to the anonymous analysis of their data at the time when they applied to participate in the contest.

### Measures and Variables – General Information

#### Leadership Emergence

Contest participants competed for peer nominations in each group task in short-term work groups. After each task, women completed a questionnaire to (a) nominate and (b) describe the three group members who had convinced them the most with respect to the group member’s potential to act as a leader. Leadership emergence was operationalized as the total number of nominations each woman received from her team members across the entire contest. The more nominations a woman received in the contest, the higher her leadership emergence index. However, we controlled for group size, that is, peer nominations possible in the contest, in the main study.

#### Ascribed Babyfacedness and Ascribed Facial Attractiveness

Raters (three in the pilot study, nine in the main study) evaluated women’s faces by watching their application videos. Raters watched a 25-s cut of one interview question without sound in order to focus solely on the participants’ outward appearance and to avoid any undue influence of other aspects (e.g., spoken content or voice). The length of the video sequence was determined on the basis of pretests as well as literature suggesting that a rating of appearance was possible after this amount of time (see Ambady and Rosenthal, 1993).

**FIGURE 1:**
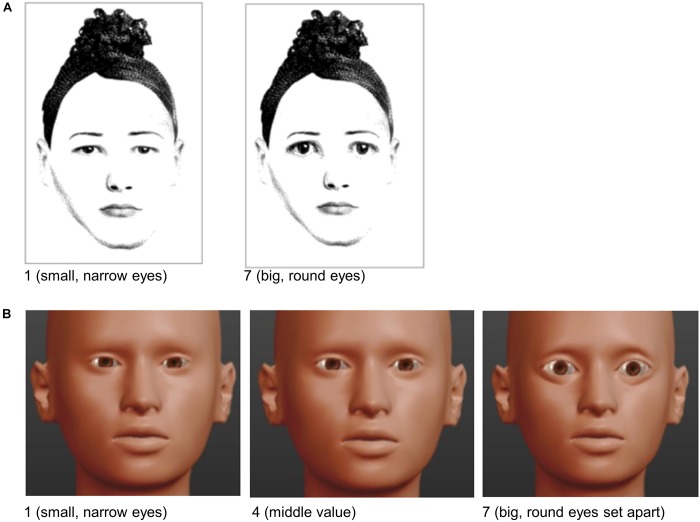
Example excerpt from the two versions of the rating manual for the babyfacedness subfacet *eyes*. **(A)** Displays the extreme values for “not babyfaced” (i.e., small and narrow eyes) in contrast to “babyfaced” (i.e., big and round eyes) from the first version of the manual. **(B)** Displays extreme values from the revised manual, including an example of mid-ranged babyfaced eyes.

After the video sequence had finished, raters assessed the women’s facial attractiveness and babyfacedness. For each babyfacedness item, a rating manual depicted example pictures for the extreme values in advance (see Figure 1 for an example of the eyes facet). The means of the attractiveness items (three in the pilot study: attractive, good looking, and pleasing; two in the main study: attractive, good looking; all ranging from 1 = low value to 10 = high value) averaged across all raters served as the predictor variable *ascribed facial attractiveness*. The predictor variable *ascribed babyfacedness* resulted of the mean ratings of the four babyfacedness items (nose, eyes, face form, overall impression, ranging from 1 = not babyfaced to 7 = very babyfaced) averaged across all raters.

Before raters gave their evaluations, they were trained on the basis of a rating manual. In the pilot study, this manual contained black-and-white pictures for the extreme values of the babyfacedness items (for an example, see Figure 1A). Before conducting the main study, the manual was revised in accordance with information obtained from the pilot study. Changing the instructions from “How would you evaluate the women shown in the videos…” to “*How would most people evaluate the women shown in the videos compared with women of the same age*?” targeted to reduce subjectivity in the raters’ impressions. The revised manual further contained higher quality pictures for the visual attributes of the babyfacedness items by using the freeware program MakeHuman (version 1.1.0, MakeHuman team, 2016) replacing lower quality pictures from the former manual version. This procedure allowed the image of detailed face avatars to represent the extreme and middle values of each item (for an example, see Figure 1B). We further revised or removed single items (see below; see the revised manual here^2^).

#### Ascribed Social Competence and Ascribed Naïveté

After having evaluated the women’s facial characteristics, the raters watched the videos (see section “Ascribed Babyfacedness and Ascribed Facial Attractiveness”) a second time, uncut and with the sound on, and estimated the women’s social competence and naïveté. The mean of all items per scale averaged across all raters served as variables further used to test for mediation effects. Ascribed social competence consisted of six items in the pilot study (sociable, confident, warm-hearted, popular, empathic, and socially competent) and of five items in the main study (verbally skilled, sociable, confident, anxious [negatively coded], socially competent; all ranging from 1 = low value to 7 = high value). Ascribed naïveté consisted of three items in the pilot study (naïve, mature [negatively coded], critical thinker [negatively coded]), and of four items in the main study (naïve, inexperienced, gullible, generally intelligent [negatively coded]; all ranging from 1 = do not agree at all to 7 = strongly agree).

#### Demographic Data

Age, professional experience, and leadership experience in years were obtained from the CVs the women submitted with their applications.

## Pilot Study

### Methods

#### Participants

In sum, 109 women who participated in the contest in 2015 (out of a total of 187 women) gave their consent to use information from their CVs and interviews for research purpose and were included. Videos of eight contest participants were excluded due to insufficient picture or sound quality. The ages of the final sample of 101 women ranged from 22 to 53 years (*M* = 31.96, *SD* = 6.68). On average, these women had 9.73 years (*SD* = 6.04) of professional experience and 6.22 years (*SD* = 4.54) of leadership experience. Furthermore, 79.2% were German citizens, 5.0% other, and 15.8% did not provide information about their nationality.

#### Materials

In the pilot study, three raters evaluated facial features and characteristics using the interview sequences from the contest application phase in 2015.^3^

#### Procedure

##### Leadership emergence

In the leadership contest, the women worked on three assessment-center-like tasks. The participants were randomly and differently assigned to groups of 10 to 12 women per each task. The challenges in the tasks were (a) to engage in a 50-min group discussion about the skills needed for successful leadership, (b) to take 80 min to build a wooden construction, including several predefined intermediate goals, and (c) to take 50 min to prepare a political speech for a female executive politician. Each participant gave written nominations of the three most convincing group peers after each task (see section “Leadership Emergence”). Leadership emergence was calculated on the basis of the General Information (see section “Leadership Emergence”).

##### Facial characteristics and ascribed traits

The ascribed facial attractiveness scale (α = 0.98), the ascribed babyfacedness scale (α = 0.78), the ascribed social competence scale (α = 0.85), and the ascribed naïveté scale (α = 0.88) all demonstrated sufficient internal consistency. The ICC values were acceptable, ranging from 0.63 to 0.82 (see section “Measures and Variables – General Information” for all items and procedure).

#### Statistical Analyses

To test Hypotheses 1–4, we calculated mediation analysis using the “lavaan” package (version 0.6-1, Rosseel, 2018) in R (version 3.5.0). In order to control for non-normality of our data, we conducted robust ULS estimators. Data and R scripts are published in the Open Science Framework (OSF^4^).

**Table 1:** Descriptive statistics for the variables for both the pilot study and the main study, presented separately by contest year.

	Pilot study		Main study (*N* = 195)			
	2015 (*N* = 101)		2016 (*N* = 111)		2017 (*N* = 84)	
	*M*	*SD*	*M*	*SD*	*M*	*SD*
Leadership emergence	9.34	5.69	6.12	3.79	4.88	3.27
Ascribed facial attractiveness [1–10]	4.98	1.72	5.92	1.27	6.00	1.38
Ascribed social competence [1–7]	4.47	0.65	4.78	0.58	4.82	0.65
Ascribed babyfacedness [1–10]	3.79	1.05	3.95	0.84	3.99	0.87
Ascribed naïveté [1–10]	3.63	0.99	3.38	0.60	3.44	0.67

*The more nominations (leadership emergence) a participant received in the contest the higher her leadership emergence. Ascription ratings were on rating scales [anchor in brackets] on which higher scores indicate a higher degree of each ascribed characteristic. N, sample size; M, mean; SD, standard deviation.*

**Table 2:** Correlations between leadership emergence (dependent variable), ascribed babyfacedness and ascribed attractiveness (predictors), and ascribed social competence and ascribed naïveté (mediators) for both the pilot study and the main study.

*r*	1	2	3	4	5
(1) Leadership emergence		0.04	0.23*	-0.10	-0.33***
(2) Ascribed attractiveness	0.12		0.52***	0.37***	0.23*
(3) Ascribed social competence	0.27***	0.20**		0.20*	-0.02
(4) Ascribed babyfacedness	-0.02	0.52***	0.07		0.26**
(5) Ascribed naïveté	-0.25***	0.13	-0.72***	0.17*	

*Higher variable scores indicate a higher degree of each ascribed characteristic. r = Pearson correlation coefficients. The values from the pilot study are presented above the diagonal, and the values from the main study (using centered variables) are presented below the diagonal. ^∗∗∗^p < 0.001, ^∗∗^p < 0.01, ^∗^p < 0.05.*

**FIGURE 2:**
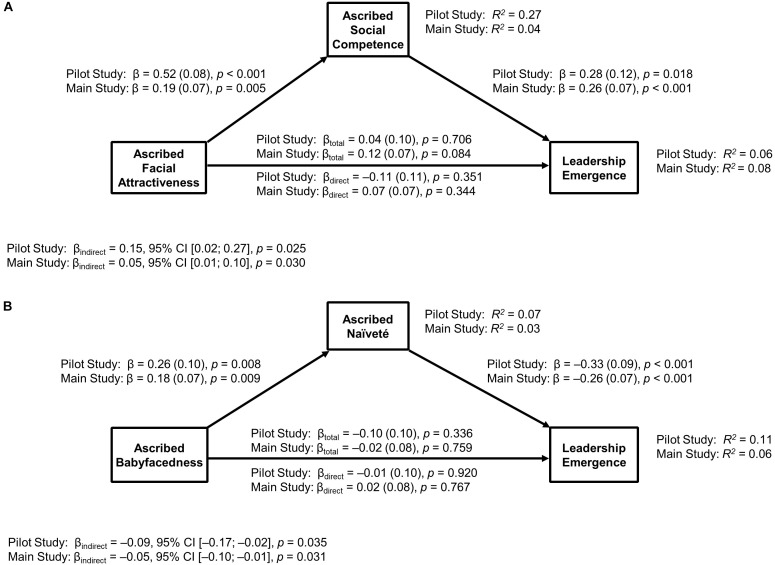
β (standard error) values represent standardized regression coefficients. _total_ = total effect. _direct_ = direct effect. *R*^2^= variance explained by the model. Results from the *pilot study* as well as from the *main study* revealed significant indirect effects for both paths, ascribed attractiveness – social competence – leadership emergence (see model **A**) as well as for ascribed babyfacedness – ascribed naïveté – leadership emergence (see model **B**).

### Results

Descriptive statistics for the dependent and independent variables are presented in Table 1, and the correlations between the variables are presented in Table 2. Results of the mediation analysis computed to test Hypotheses 1 and 2, displayed in Figure 2A, revealed that ascribed facial attractiveness was a significant predictor of ascribed social competence (β = 0.52, *SE* = 0.08, *p* < 0.001) and that ascribed social competence was a significant predictor of leadership emergence (β = 0.28, *SE* = 0.12, *p* = 0.018). A bootstrapping estimation approach with 1,000 bootstrapped samples indicated a significant indirect effect (β_indirect_ = 0.15, 95% CI [0.02, 0.27], *p* = 0.025). Yet, ascribed facial attractiveness did not significantly predict leadership emergence before (β_total_ = 0.04, *SE* = 0.10, *p* = 0.706) or after (β_direct_ = -0.11, *SE* = 0.11, *p* = 0.351) adding the mediator, ascribed social competence. Because there was a significant indirect effect (i.e., the higher a contest participant’s ascribed facial attractiveness, the higher her ascribed social competence; and the higher her ascribed social competence, the higher her leadership emergence), we concluded that ascribed social competence acted as a mediator between ascribed facial attractiveness and leadership emergence, even though the analysis did not reveal a significant total effect.^5^

The results of the mediation analysis for Hypotheses 3 and 4 indicated that ascribed babyfacedness was a significant predictor of ascribed naïveté (β = 0.26, *SE* = 0.10, *p* = 0.008) and that ascribed naïveté was a significant predictor of leadership emergence (β = -0.33, *SE* = 0.09, *p* < 0.001). The bootstrapping estimation approach with 1,000 bootstrapped samples indicated a significant indirect effect (β_indirect_ = -0.09, 95% CI [-0.17, -0.02], *p* = 0.035). Ascribed babyfacedness did not significantly predict leadership emergence before (β_total_ = -0.10, *SE* = 0.10, *p* = 0.336) or after adding the mediator, ascribed naïveté (β_direct_ = -0.01, *SE* = 0.10, *p* = 0.920). The results indicated a mediating effect of ascribed naïveté, which means that the higher a participant’s ascribed babyfacedness, the higher her ascribed naïveté; and the higher her ascribed naïveté, the lower her leadership emergence, as Figure 2B illustrates.

## Main Study

### Methods

#### Participants

We included data from a total of 195 contest participants from 2016 (111 of the 178 participants gave consent) and 2017 (84 of the 114 participants gave consent) in our main study. The ages of the contest participants ranged from 21 to 52 (*M* = 32.81, *SD* = 6.69). On average, these women had 9.61 years (*SD* = 6.12) of professional experience and 4.69 years (*SD* = 4.33) of leadership experience (for information separated by year, see Table 3). In 2016, 75.6% were German citizens, 2.4% other, and 22% did not provide information about their nationality. In 2017, 18.2% were German citizens, 9.1% other, and 71.7% did not provide information about their nationality.

#### Materials

For the 2016 and 2017 samples used in the main study, nine raters analyzed the contest participants’ self-recorded video interviews.^6^

#### Procedure

##### Leadership emergence

In the leadership contests in 2016 and 2017, in groups that were each comprised of 7 to 10 women, the women worked on three assessment-center-like group tasks in 2016 and on only two tasks in 2017. We excluded one of the tasks used in the 2016 contest from our analysis to enhance comparability between the two samples. In 2016, the challenges in the tasks were (a) to engage in a 45-min group discussion and (b) to spend 40 min preparing an industrial lobby presentation. In 2017, the women were asked (c) to spend 60 min preparing a huge event and (d) to spend 60 min preparing for a public debate on the gender issue. Each participant provided written nominations of the three most convincing group peers after each task (see section “Leadership Emergence”). We summed the number of nominations for each participant. For the analyses, we divided this sum of nominations by the number of peer nominations possible (i.e., group size per task minus one) in order to control for contaminations due to effects of group size.

##### Facial characteristics and ascribed traits

In the main study, the ascribed facial attractiveness scale (α = 0.98), the ascribed babyfacedness scale (α = 0.82), the ascribed social competence scale (α = 0.93), and the ascribed naïveté scale (α = 0.90) all demonstrated sufficient internal consistency. ICC values for all ratings in the main study ranged from 0.88 to 0.89 for the ascribed facial attractiveness items, from 0.81 to 0.89 for the ascribed babyfacedness items, from 0.71 to 0.84 for the ascribed social competence items, and from 0.68 to 0.83 for the ascribed naïveté items (see section “Measures and Variables – General Information” for all items and the procedure).

#### Statistical Analyses

Before we calculated the mediation analyses in a manner that was similar to the analyses used in the pilot study, we centered the predictor, mediator, and dependent variables on the mean of the respective contest year in order to enhance comparability for the total sample in the main study. For example, a participant’s ascribed facial attractiveness may have been high in comparison with the entire female population but not in comparison with her competitors in the contest, if all contest participants were also rated as highly attractive. By centering the variables on the respective sample means, we corrected for such potential group characteristics.

As we did in the pilot study, we ran mediation analyses using the “lavaan” package in R. Seven participants had to be excluded from these analyses due to missing information on group size. We further controlled for possible issues of non-normality by, again, using robust ULS estimators. To control for the high correlations between the predictor variables ascribed facial attractiveness and ascribed babyfacedness as well as between the mediator variables, ascribed social competence and ascribed naïveté, revealed by the a priori analyses, we ran further analyses in which we included all variables simultaneously in one path model. As postulated by Preacher and Hayes (2008), this procedure allowed to identify the unique capacity of each mediator to account for the impact of the respective predictor on leadership emergence beyond the shared contribution of the two mediators. Data and R scripts are published in the OSF^7^.

**Table 3:** Age and years of professional and leadership experience of women in the main study sample, presented separately by contest years and overall.

Main study sample		2016	2017	overall

Sample size	*N*	111	84	195
Age	*M (SD*)	33.39 (6.98)	30.20 (4.48)	32.81 (6.69)
	Min – Max	21–52	24–42	21–52
Professional experience in years	*M (SD*)	10.07 (6.31)	8.43 (5.48)	9.61 (6.12)
Leadership experience in years	*M (SD*)	4.61 (4.48)	4.99 (3.83)	4.69 (4.33)

*N, sample size; M, mean; SD, standard deviation.*

### Results

Contest participants in the main study received an average of 5.58 nominations (*SD* = 3.62) across the two tasks. On average (and before the variables were centered), the mean of ascribed facial attractiveness was 5.96 (*SD* = 1.31), and the mean of ascribed social competence was 4.80 (*SD* = 0.61). The mean of ascribed babyfacedness was 3.97 (*SD* = 0.85), and the mean of ascribed naïveté was 3.41 (*SD* = 0.63). See Table 1 for values separated by contest year, and Table 2 for intercorrelations between the variables.

As hypothesized, the data analyses indicated that ascribed social competence mediated the influence of ascribed facial attractiveness on leadership emergence. Confirming the results of the pilot study, the higher a contest participant’s ascribed facial attractiveness, the higher her ascribed social competence (β = 0.19, *SE* = 0.07, *p* = 0.005), and the higher her ascribed social competence, the higher her leadership emergence (β = 0.26, *SE* = 0.07, *p* < 0.001). The bootstrapping estimation approach with 1,000 bootstrapped samples indicated a significant indirect effect (β_indirect_ = 0.05, 95% CI [0.01, 0.10], *p* = 0.030). However, ascribed facial attractiveness did not significantly predict leadership emergence, neither before (β_total_ = 0.12, *SE* = 0.07, *p* = 0.084) nor after adding the mediator, ascribed social competence (β_direct_ = 0.07, *SE* = 0.07, *p* = 0.344; see Figure 2A).

The second mediation analysis revealed that ascribed naïveté functioned as a mediator of the influence of ascribed babyfacedness on leadership emergence. According to our results, ascribed babyfacedness significantly predicted ascribed naïveté (β = 0.18, *SE* = 0.07, *p* = 0.009). Further, ascribed naïveté significantly predicted leadership emergence (β = -0.26, *SE* = 0.07, *p* < 0.001), supporting the results of the pilot study as well. In other words, the higher a participant’s ascribed babyfacedness, the higher her ascribed naïveté. However, the higher her ascribed naïveté, the lower her leadership emergence. The bootstrapping estimation approach with 1,000 bootstrapped samples indicated a significant indirect effect (β_indirect_ = -0.05, 95% CI [-0.10, -0.01], *p* = 0.031). Further, in line with the results of the pilot study, ascribed babyfacedness did not predict leadership emergence, neither before (β_total_ = -0.02, *SE* = 0.08, *p* = 0.759) nor after adding the mediator, ascribed naïveté (β_direct_ = 0.02, *SE* = 0.08, *p* = 0.767; see Figure 2B).

**FIGURE 3:**
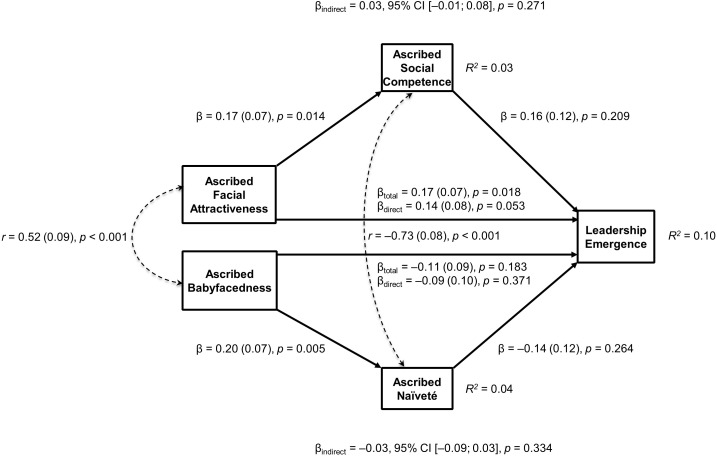
Testing both mediation hypotheses in one path model [χ^2^(2, *N* = 188) = 0.43, CFI = 1.00, RMSEA < 0.001, SRMR = 0.012] in the main study sample with 1,000 bootstrapping samples. β (standard error) values represent standardized regression coefficients. _total_ = unique total effect. _direct_ = unique direct effect. _indirect_ = unique indirect effect. Unique indirect effects were not significant for both paths ascribed attractiveness – ascribed social competence – leadership emergence as well as for ascribed babyfacedness – ascribed naïveté – leadership emergence.

We further tested the unique ability of each mediator to mediate the corresponding relation between the predictor and leadership emergence in one joint path model. In this model, we controlled for the correlations between the predictor variables and the mediator variables in order to identify the unique impact of each mediator. Overall, the model showed a very good fit, χ^2^(2, *N* = 188) = 0.43, CFI = 1.00, RMSEA < 0.001, SRMR = 0.012, and is displayed in Figure 3 with all individual regression coefficients. The unique indirect effects were all non-significant. More specifically, neither the effect of ascribed facial attractiveness on leadership emergence through ascribed social competence (β_indirect_ = 0.03, 95% CI [-0.01, 0.08], *p* = 0.271), nor the effect of ascribed facial babyfacedness on leadership emergence through ascribed naïveté (β_indirect_ = -0.03, 95% CI [-0.09, 0.03], *p* = 0.334) reached the conventional thresholds of significance. Furthermore, there were no unique direct effects of ascribed facial attractiveness (β_direct_ = 0.14, *SE* = 0.08, *p* = 0.053), ascribed babyfacedness (β_direct_ = 0.09, *SE* = 0.10, *p* = 0.371), ascribed social competence (β = 0.16, *SE* = 0.12, *p* = 0.209), or ascribed naïveté (β = -0.14; *SE* = 0.12, *p* = 0.264) on leadership emergence. However, when we combined the unique direct and indirect effects of ascribed facial attractiveness, we found a significant unique total effect on leadership emergence (β_total_ = 0.17, *SE* = 0.07, *p* = 0.018), suggesting that ascribed facial attractiveness had an effect on leadership emergence above and beyond ascribed babyfacedness and ascribed naïveté.

## General Discussion

Aim of the present study was to increase the understanding of the effects of facial appearance and ascribed characteristics on leadership emergence by employing data collected at a women’s leadership contest. We captured characteristics of facial appearance by rating participants’ application videos as they responded to standardized interview questions. Peer nominations of the women from two exercises that were part of the contest served as a measure of leadership emergence. Analyses of data from both a pilot study and a main study revealed a significant indirect effect of ascribed facial attractiveness and babyfacedness ratings made by independent raters on leadership emergence. Ascribed social competence mediated the relation between ascribed facial attractiveness and leadership emergence, and ascribed naïveté mediated the relation between babyfacedness and leadership emergence. However, the results did not support Hypotheses 1 and 3 because neither ascribed facial attractiveness nor ascribed babyfacedness significantly predicted leadership emergence. When we tested the hypotheses separately, these findings held both, independent from the inclusion or exclusion of the mediators. Therefore, the analyses in this study revealed that both the women’s perceived attractiveness and their babyfacedness were not directly related to their leadership emergence but had an impact through ascribed aspects that were explicitly or implicitly related to these characteristics.

Following the recommendations of a reviewer, we also tested the unique effects of our two mediators and predictors on leadership emergence. Analyses revealed a significant unique effect on leadership emergence only for ascribed facial attractiveness after controlling for ascribed babyfacedness and ascribed naïveté. This finding suggests that the previously reported indirect effects of ascribed social competence and ascribed naïveté showed a large overlap of variance and that the shared and not the unique portion of ascribed personality traits variance mediated the effect of ascribed facial appearance on leadership emergence. Therefore, this finding could signify that an underlying latent variable, namely, ascribed leadership personality, is mediating the effect between ascribed facial appearance and leadership emergence. However, our study was not equipped to handle such a latent variable model due to an insufficient sample size and number of indicators. Future research should investigate the unique and common portions of indirect effects of ascribed facial appearance on leadership emergence through ascribed personality traits by including more indicators for ascribed facial appearance and ascribed personality traits.

These findings also suggest a higher relevance of facial attractiveness for leadership emergence because babyfaced women were perceived as more attractive. However, literature indicated babyfacedness to be related to attractiveness, especially in women (Zebrowitz McArthur and Apatow, 1983/1984). The model employed cannot be used to determine such a halo effect (i.e., possessing babyface causing higher attractiveness ratings in women) because we had defined attractiveness and babyfacedness as equal predictors. In the rating procedure, raters judged facial attractiveness directly before assessing babyfacedness; thus, the latter could not have influenced the attractiveness ratings directly. However, the results of the joint model could further indicate a compensation for the negative influence of babyfacedness on leadership emergence at the same time: In the joint model, partialling out the variance of babyfacedness may explain the significant effect of facial attractiveness on leadership emergence. Babyfacedness has been described as being detrimental to leadership emergence (Cherulnik et al., 1990), but also as positively correlated with facial attractiveness in women (Zebrowitz McArthur and Apatow, 1983/1984). After controlling for babyfacedness in facial attractiveness, the negative effect of babyfacedness via facial attractiveness was no longer relevant. Thus, the ‘pure’ facial attractiveness revealed an effect on leadership emergence (i.e., without the confounding effect of babyfacedness). Facial attractiveness was thus no longer influenced by babyfacedness but by other characteristics as, for instance, the symmetry, healthy appearance and other aspects, which we did not explicitly assess.

Further bearing in mind the relation between babyfaced features and femininity (Friedman and Zebrowitz, 1992), the question arises as to whether higher babyfacedness (and thus higher attractiveness) accents the feminine gender role in women and, hence, increases discrimination against them (Zebrowitz et al., 1991). Concluding from our results revealing that babyfacedness weakened the positive effect of attractiveness, it seems that the part of women’s attractiveness associated with femininity is not the one promoting but rather preventing their leadership emergence. Further, would babyfaced women therefore have to increase their agentic behaviors to compensate for their even more accentuated gender role in order to emerge as leaders, as indicated by role congruity theory (see Eagly and Karau, 2002; Eagly and Carli, 2007)? Because features of appearance and accordingly ascribed traits depict only some relevant aspects that predict leadership emergence, future research should also include measures of behaviors and other personality traits, for instance, perceived agency and communion (Johnson et al., 2008; Schock et al., 2018) or task-specific aspects (e.g., Eagly and Karau, 1991; Badura et al., 2018).

Ascribed social competence, related to facial attractiveness, indicated an advantage for obtaining a large number of nominations in the women’s leadership contest. On the other hand, ascribed naïveté, related to babyfacedness, indicated a disadvantage. The socially enhancing effects of aspects of attractiveness were well in line with the literature (e.g., Eagly et al., 1991; Langlois et al., 2000). Moreover, a childlike appearance and ascribed naïveté represent characteristics that are counter to the characteristics typically ascribed to leaders (e.g., Zebrowitz McArthur and Apatow, 1983/1984; Zebrowitz et al., 1991; Friedman and Zebrowitz, 1992; Masip et al., 2004; Riggio and Riggio, 2010).

These results are particularly remarkable because the predictor and criterion variables were assessed with different methods. Although both kinds of variables were assessed with ratings, the ratings were made by different samples: The raters of the videos were students, whereas the raters in the contest were competing peers. Furthermore, the predictor variables consisted of ratings of recorded video interviews, whereas the criterion variables based upon real-life interactions in a competitive situation. As the two kinds of raters may have perceived the same targets of rating in different contexts, somewhat different processes – or at least differing on the level of awareness – may have taken place. For instance, the participants of the contest may have focused more on the competition and their own performance, whereas the raters in the lab explicitly concentrated on the features of facial appearance and personality they had to assess. The finding that ratings of video recordings were related to ratings from a real-life situation may hint at two possible mechanisms: First, the impressions that the raters got from the video recordings were similar to the impressions that the peers had during the contest, the latter of which may have shaped the interactions and ascriptions to a certain extent (see Antonakis and Eubanks, 2017). Therefore, the results may suggest that the ascribed social competence as well as the ascribed naïveté of the women may reflect the important and consensual information that was relevant for raters in both situations (i.e., the application and the contest).

Nevertheless, our results are well in line with previous research on the relevance of appearance for election outcome predictions in political leadership (e.g., Todorov et al., 2005; Antonakis and Dalgas, 2009). In other words, ratings of aspects of physical appearance made by individuals who are not voting in the actual leader election (or as in our study, in the leadership contest) could in part predict the outcome of a leader election (or as in our study, a leader competition).

Second and alternatively, these results may also indicate that consistency in first impressions based on facial appearance may lead people to treat other individuals in certain ways that subsequently shape these individuals’ outcomes and behaviors (see Rule and Ambady, 2010; Lukaszewski and Roney, 2011). Further, such impressions and associations based on facial appearance may be extended to the interactional mechanism that underlies a self-fulfilling prophecy (Snyder et al., 1977; further, see Zebrowitz and Montepare, 2008; Todorov et al., 2015). Acting on the assumption that the nominations made by the peers in the contest as well as the video ratings made by external raters reveal real aspects of the participants, according to the self-fulfilling prophecy mechanism, the participants may have developed their characteristics (e.g., social competence or naïveté) on the basis of how they were treated due to their facial appearance, attractiveness, and babyfacedness, respectively. Analogously, at the contest, women may have treated an attractive peer who they therefore perceived as socially competent in a way that increased the peer’s confidence in acting like a leader. This, in turn, may have led to more actual leadership behavior, thus convincing the other contestants to nominate her as a convincing leader. For example, the fact that attractiveness may have served as a leadership cue may have led participants to pay more attention to particular women in the contest, which in turn may have increased the abilities of these particular women to excel in the group task (see Gerpott et al., 2017).

Another advantage of this study is that the data collection was part of a real contest, and the sample consisted of real women who participated in the study to win the contest and enhance their reputations. This competitive setting could be interpreted as comparable to an actual personnel selection situation (e.g., an assessment center) where performance is required within a short period of time. However, the rather unconventional study setting somewhat restricts the interpretation with regard to generalizability: Peers from group tasks usually do not make hiring decisions in personnel selection, but rather, external observers or assessment experts do. Nevertheless, we aimed to contribute to knowledge about which variables are relevant for leadership emergence. Conclusions cannot be drawn with respect to leadership ascription or leadership performance in the medium or long term, especially in comparison with day-to-day teamwork without a competitive character, even though the contest participants had to balance competitiveness with cooperation in working toward a common goal during the tasks. However, our research revealed that appearance is especially important when no information or only a little other information is available (Olivola et al., 2012). In line with previous research, we would expect the impact of facial appearance on leader selection to decrease when information about competence is available because competence-related information should have a greater impact on selection decisions (Kaufmann et al., 2017). Although facial appearance had an impact on women’s chances of getting more nominations in the leadership contest in this study, we would expect this effect to diminish in the medium and long terms after women have entered the work place.

Although the setting allowed obtaining an unusual sample of women, future research should include male participants as well as other ethnicities (e.g., Blacks) to investigate interactions between sex and other personal attributes. For instance, previous research indicated that attractiveness judgments were better predictors of election success for women than for men (Berggren et al., 2010). Furthermore, whereas the current study indicated a disadvantage for individuals possessing facial physiognomy reflecting babyfacedness, Livingston and Pearce (2009), for example, found that a larger number of black male CEOs had babyfaced characteristics in their study compared with white male CEOs. A newer study addressing political voting decisions in an Asian culture revealed that babyfacedness was the strongest predictor of percentages of votes beyond competence, attractiveness, warmth, and background characteristics (Chang et al., 2017).

Our findings provide support for earlier studies (e.g., Sczesny and Kühnen, 2004; Olivola and Todorov, 2010; Re and Perrett, 2014) that found that aspects of physical appearance such as facial attractiveness and babyfacedness affect the attribution of job-relevant characteristics in women. This effect may also play an important role in personnel selection. Future studies should investigate the particular relevance of physical appearance with regard to job profiles and job domains. Furthermore, future studies should investigate how other aspects of physical appearance (e.g., body size) impact this effect and should further investigate strategies for overcoming possible disadvantages related to facial appearance, for example, via clothing or hairstyle.

Visual appearance often influences the first impression a person makes, especially in personnel selection (e.g., based on photographs in CVs) and therefore affects the attributions made to an individual. Under a more practical perspective, we encourage organizations and hiring experts to enhance their awareness of such ascription- and stereotype-inducing mechanisms, helping women to overcome these gender-related obstacles to achieve leadership positions equally to their male counterparts. The use of standardized assessment criteria that are determined in advance (from the side of human resources) and the decision not to include a photo in a job application (from the side of an applicant) may be first steps to diminish such effects, at least with regard to aspects of (facial) appearance.

## Data Availability

Datasets are in a publicly accessible repository: the datasets analyzed for this study can be found in the Open Science Framework (https://osf.io/kxm4w/).

## Ethics Statement

This study was carried out in accordance with the recommendations of the statutes of the University of Salzburg (part XI), Ethic Committee of the Paris Lodron University of Salzburg. The protocol was approved by the Ethic Committee of the Paris Lodron University of Salzburg. All subjects gave written informed consent in accordance with the Declaration of Helsinki.

## Author Contributions

FG collected the data for the pilot study, co-revised the rating manual, and supervised and organized the data collection and analyses for the main study. She mainly contributed to writing the Method, Results, and Discussion sections of the manuscript and revised earlier drafts of the entire manuscript. CV conceptualized the research, data collection and analyses of both the pilot study and the main study. She mainly contributed to writing the first versions of the Introduction and Discussion sections as well as the Results section of the pilot study. TO supervised the conceptualization of this research regarding the operationalization and methods. She revised earlier drafts of the manuscript, in particular providing theoretical input for the Introduction and Discussion sections.

## Conflict of Interest Statement

The authors declare that the research was conducted in the absence of any commercial or financial relationships that could be construed as a potential conflict of interest.

## Footnotes

1 Visit www.we-are-panda.com for more information about the contests.

2 https://osf.io/sf72x/

3 Detailed interview questions can be obtained from the first author.

4 https://osf.io/kxm4w/

5 Counter to previous standards (see Baron and Kenny, 1986), researchers have suggested that it is acceptable to interpret a mediation effect on the basis of a significant indirect effect even in the absence of a significant direct or total effect (e.g., MacKinnon et al., 2007; Hayes, 2018).

6 Detailed interview questions can be obtained from the first author.

7 https://osf.io/kxm4w/
